# Inspiratory and expiratory resistance cause right‐to‐left bubble passage through the foramen ovale

**DOI:** 10.14814/phy2.13719

**Published:** 2018-06-27

**Authors:** Kayla L. Moses, McKayla Seymour, Arij Beshish, Kim R. Baker, David F. Pegelow, Luke J. Lamers, Marlowe W. Eldridge, Melissa L. Bates

**Affiliations:** ^1^ John Rankin Laboratory of Pulmonary Medicine Department of Pediatrics Critical Care Division University of Wisconsin School of Medicine and Public Health Madison Wisconsin; ^2^ Department of Kinesiology University of Wisconsin‐Madison Madison Wisconsin; ^3^ Department of Health and Human Physiology University of Iowa Iowa City Iowa; ^4^ Adult Echocardiography Laboratory University of Wisconsin Hospitals and Clinics Madison Wisconsin; ^5^ Division of Pediatric Cardiology University of Wisconsin School of Medicine and Public Health Madison Wisconsin; ^6^ Department of Biomedical Engineering University of Wisconsin‐Madison Iowa City Iowa; ^7^ Stead Family Department of Pediatrics University of Iowa Iowa City Iowa; ^8^ Holden Comprehensive Cancer Center University of Iowa Iowa City Iowa

**Keywords:** Airway resistance, diving, patent foramen ovale, shunt, stroke, work of breathing

## Abstract

A patent foramen ovale (PFO) is linked to increased risk of decompression illness in divers. One theory is that venous gas emboli crossing the PFO can be minimized by avoiding lifting, straining and Valsalva maneuvers. Alternatively, we hypothesized that mild increases in external inspiratory and expiratory resistance, similar to that provided by a SCUBA regulator, recruit the PFO. Nine healthy adults with a Valsalva‐proven PFO completed three randomized trials (inspiratory, expiratory, and combined external loading) with six levels of increasing external resistance (2–20 cmH_2_O/L/sec). An agitated saline contrast echocardiogram was performed at each level to determine foramen ovale patency. Contrary to our hypothesis, there was no relationship between the number of subjects recruiting their PFO and the level of external resistance. In fact, at least 50% of participants recruited their PFO during 14 of 18 trials and there was no difference between the combined inspiratory, expiratory, or combined external resistance trials (*P* > 0.05). We further examined the relationship between PFO recruitment and intrathoracic pressure, estimated from esophageal pressure. Esophageal pressure was not different between participants with and without a recruited PFO. Intrasubject variability was the most important predictor of PFO patency, suggesting that some individuals are more likely to recruit their PFO in the face of even mild external resistance. Right‐to‐left bubble passage through the PFO occurs in conditions that are physiologically relevant to divers. Transthoracic echocardiography with mild external breathing resistance may be a tool to identify divers that are at risk of PFO‐related decompression illness.

## Introduction

The foramen ovale is an atrial septal shunt present that functionally closes at birth. Divers with a patent foramen ovale (PFO) have a four‐time greater prevalence of decompression illness (DCI) than divers without a PFO (Torti et al. 2004). Intra and extravascular nitrogen microbubbles, the hallmark of DCI, are formed secondary to reductions in atmospheric pressure occurring on diving ascents, as the solubility of dissolved gases within the plasma and tissues decrease. Consequences of DCI can include nitrogen microbubble formation within tissues to cause local damage (decompression sickness), pulmonary barotrauma, and injury due to systemic‐arterial gas emboli (Moon et al. 1995). Thus, diving tables and computers monitoring depth, pressure, compression duration, and rate of ascent, are strictly recommended to ensure that microbubble formation is maintained at a rate in which the pulmonary capillaries can effectively capture and dissipate microbubbles via alveoli. However, despite adequate precautions, roughly half of all cases of DCI are suffered by divers who had performed safe diving profiles, leaving the onset of their DCI unexplained (Moon et al. 1995). Right‐to‐left passage of bubbles through the PFO may allow bubbles to bypass the pulmonary circulation and contribute to DCI.

After birth, the pulmonary vasculature dilates, ultimately resulting in a decrease in right atrial pressure, relative to the left atrial pressure (Anderson et al. 2002). The reversal of the atrial pressure gradient causes the flap‐like opening of the foramen ovale to functionally close against the atrial septal wall. Fibrous adhesions develop to anatomically close the PFO in most individuals, but this closure is incomplete in 20–50% of adults (Patten 1938; Hagen et al. 1984; Fisher et al. 1995; Elliott et al. 2013; Marriott et al. 2013). In the absence of anatomical closure, an increase in right‐to‐left atrial pressure gradient temporarily opens the foramen ovale, and allow for right‐to‐left flow of blood and bubbles.

We previously studied the frequency of foramen ovale recruitment in response to changes in inspired oxygen concentration and body position (Moses et al. 2015). Unexpectedly, 45% of participants with a recruited their PFO during quiet breathing through a low resistance mouthpiece, typically used for exercise physiology studies. The mouthpiece used during trials increased inspiratory and expiratory breathing resistance by 1.5 cmH_2_O/L/sec. While this change in resistance is small, we speculated that it may have been sufficient to change intrathoracic pressure, thereby increasing venous return and increasing right atrial pressure. This could mechanistically explain the foramen ovale recruitment in our participants. When the Valsalva is used clinically to detect a PFO, the increased intrathoracic pressure collapses the inferior vena cava, increasing the venous pressure behind the site of collapse. When the maneuver is released, there is a brief increase in the venous return, which raises the right atrial pressure. Similarly, we speculated that added external breathing resistance causes pulsatile increases in venous return through a similar mechanism, by compressing the vena cava and allowing transient increases in venous return during inhalation (Olgiati et al. 1986). However, given our previous study design, it is impossible to determine whether external breathing resistance changes the intrathoracic pressure and whether this is associated with recruitment of the foramen ovale.

The resistance provided by diving regulators depends on the design of the regulator itself, and also on the temperature of the water and atmospheric pressure. It has been experimentally determined that resistances less than 20 cmH_2_O/L/sec are generally tolerable (Warkander et al. 2001). Given our previous findings, and the current lack of knowledge as to whether external breathing resistance recruits the foramen ovale, we sought to determine if increasing breathing resistance within this tolerable range contributes to a PFO. We examined incrementally increased inspiratory, expiratory, and combined inspiratory and expiratory resistance in a population of healthy adults with a previously proven, Valsalva‐induced PFO. We hypothesized that the frequency with which individuals recruit their foramen ovale would increase as a function of increased external resistance, secondary to an increase in esophageal pressure, which is an index of intrathoracic pressure. Three randomized trials (inspiratory, expiratory, and combined) consisting of six levels of incrementally increasing resistance, to a maximum resistive load of 20 cmH_2_O/L/sec, were performed in nine participants with a PFO. Contrary to our hypothesis, we found that the frequency of observed PFO in our population did not correlate with resistance or intrathoracic pressure. Instead, we found that the rate of PFO recruitment depended on the individual, with some individuals having particularly high or low rates of recruitment regardless of the resistance.

## Materials and Methods

### Subjects

Thirty‐four (16 female) healthy, nonsmoking adults aged 18–35 years were recruited from the University of Wisconsin‐Madison and surrounding community. Exclusion criteria included current pregnancy or breastfeeding, previous diagnosis of cardiopulmonary disease, daily medication use other than hormonal birth control, and any neurological or motor deficit that would prevent participation. The study received approval from the University of Wisconsin School of Medicine and Public Health's Institutional Review Board. Each subject gave written, informed consent prior to participating.

### Screening for the presence of PFO

Saline contrast echocardiography, with and without a Valsalva maneuver, was used to identify the presence of PFO among participants. We have previously described the standardized PFO screening procedure used by our laboratory (Bates et al. 2014; Moses et al. 2015). Briefly, a 22‐gauge catheter was inserted in an antecubital vein and externally connected to two, three‐way stopcocks attached in series. A 10 mL syringe was attached to each stopcock. Agitated saline contrast bubbles were created by manually flushing 4 mL of sterile saline and 1 mL of air between the two syringes. The Valsalva maneuver was standardized such that each participant wore nose clips and exhaled against an occluded mouthpiece to generate +40 cmH_2_O mouth pressure, coinciding with the injection of agitated saline contrast. A four‐chamber apical view of the heart was obtained. Pressure was released upon observation of bubbles in the right atrium and the trans‐septal passage of bubbles was assessed (Vivid i, GE Ultrasound). All participants had a transient, leftward bowing of the atrial septum after release of the Valsalva. This procedure was repeated without a Valsalva maneuver, while participants rested in the left lateral decubitus position. The occurrence of atrial level bubble passage without a Valsalva maneuver is either indicative of a foramen ovale that is recruited by contrast injection alone, or of an atrial septal defect. In order to minimize the likelihood that we would include participants that had an atrial septal defect or intrapulmonary shunt, we set exclusion criteria as trans‐septal contrast passage without a Valsalva or delayed appearance of left heart contrast without a Valsalva maneuver, indicative of an intrapulmonary shunt. Finally, the atrial and ventricular septa were imaged in a subcostal view with 2D color‐flow Doppler to further exclude atrial and ventricular septal defects. Screening spirometry was also performed using previously described methods (Bates et al. 2014; Farrell et al. 2015) in order to verify that all subjects had clinically normal pulmonary function (FEV_1_ > 80% predicted, FEV_1_/FVC >0.70).

Of the 34 individuals screened, 13 were positive for a Valsalva‐induced PFO. However, three of these individuals had delayed left heart contrast at rest, consistent with intrapulmonary shunting (Elliott et al. 2013) and one had delayed atrial‐level contrast passage without Valsalva. The remaining nine individuals with a Valsalva‐induced PFO were included in this study. The average bubble score was 3, (Range 2–4) with only one subject having a grade 4 PFO (Moses et al. 2015).

### Esophageal pressure monitoring

The nose, pharynx, and throat were numbed with benzocaine spray and 2% lidocaine gel. Intrathoracic pressure was estimated using a 10‐cm latex esophageal balloon catheter (Cooper Surgical; Trumbull, CT) placed in the lower third of the esophagus. The catheter was connected to a Validyne transducer (model MP45‐1, 0–200 cmH_2_O range, Northwood, CA), and pressure waveforms were recorded (LabChart, AD Instruments, Colorado Springs, CO). The participant stood and the catheter was advanced and then withdrawn until end expiratory esophageal pressure was negative, but only minimal cardiac waveform was noted.

### External breathing resistance

Subjects initially breathed through a mouthpiece and nonrebreather valve assembly (Model 2700, Hans Rudolph) that offered a trivial amount of external resistance (<0.1 cmH_2_O/L/sec). Custom‐built mesh screens were then inserted in the inspiratory, expiratory, or both sides of the assembly in order to increase external breathing resistance. The resistance provided by the screens ranged from 2 to 20 cmH_2_O/L/sec. These resistances were determined experimentally by measuring the pressure drop across the screens with a controlled flow of room air = 1 L/sec. Resistance levels were chosen to achieve but not exceed the published values of maximum tolerable inspiratory and expiratory resistance (20 cmH_2_O/L/sec), when breathed for 25 min at the greatest depth the standard U.S. Navy decompression table allows (depth equivalent of 6.8 ATM) (Warkander et al. 2001).

### Experimental protocol

The order of the three breathing trials, inspiratory, expiratory, and combined inspiratory and expiratory resistance was randomized, but increases in resistance within each trial occurred incrementally. Participants initially breathed against no resistance, then resistance was increased 2–4 cmH_2_O/L/sec every 3 min, until a maximum resistance of 20 cmH_2_O/L/sec. Saline contrast echocardiography was performed during the last 30 sec of each level, and the trans‐atrial passage of bubbles was assessed over 20 cardiac cycles following right heart opacification.

### Data analysis

Data are displayed as mean ± standard deviation, unless otherwise indicated. Three individuals trained in interpreting echocardiography independently evaluated the echocardiograms. This included a licensed cardiac and vascular sonographer (KRB) and a clinical cardiologist (LJL), both of whom were blinded to the study design and hypothesis until the analysis was complete. A repeated measures ANOVA was used to compare the frequencies of PFO recruitment between inspiratory, expiratory, and combined resistance trials (using Tukey's HSD method for multiple comparisons). *P*‐values for two‐sided statistical tests were considered statistically significant at <0.05 (SAS version 9.4, Cary, NC). To evaluate whether maximal, minimal, and the change in esophageal pressure were related to PFO recruitment, a repeated measures, general linear model was created in which PFO patency and the individual were included as random variables, and the resistance level was treated as a covariate. A factor was considered significant when *P *< 0.05. (Minitab 18, State College, PA).

## Results

**Figure 1:**
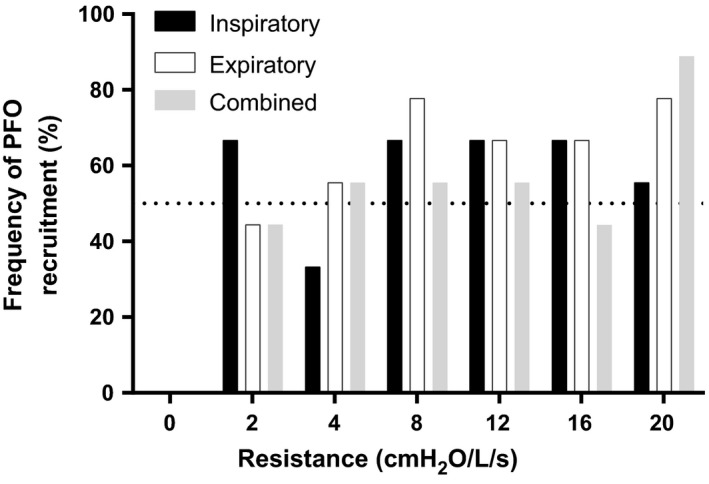
Percent frequency of patent foramen ovale (PFO) recruitment at each level (0–20 cmH_2_O) of inspiratory, expiratory, and combined external resistance. No subject recruited their PFO with 0 cmH_2_O external resistance. At least 50% of participants recruited their PFO during 14 of the remaining 18 stages (2–20 cmH_2_O).

Anthropometric characteristics and pulmonary function data are shown in Table 1. Subjects were not obese and had clinical normal lung function. No PFO recruitment was observed when subjects breathed through the mouthpiece alone, which added only a trivial amount of resistance. The addition of external resistance caused PFO recruitment, with more than half of subjects recruiting their PFOs in 14/18 trials (Fig. 1). However, there were no significant differences in the frequency of PFO observation between trials or as a function of increasing resistance. That is to say, contrary to our hypothesis, the frequency of PFO recruitment did not increase with increases in resistance.

**Table 1:** Anthropometric and pulmonary function data (*n* = 3 male, *n* = 6 female)

Age, yr	25 ± 5
Height, cm	175.3 ± 7.6
Weight, kg	76.2 ± 11.1
BMI, kg/m^2^	24.8 ± 3.9
FVC, L	5.1 ± 1.3
FEV_1_, L	4.1 ± 1.3
FEV_1_, % predicted	107.0 ± 16.3
FEV_1_/FVC, %	79.2 ± 9.2

Values are mean ± SD. yr, years; BMI, body mass index; FVC, forced vital capacity; FEV_1_, forced expiratory volume in 1 sec.

We evaluated the impact of breathing resistance on cardiovascular and ventilatory variables (Table 2). Increasing external resistance did not cause significant changes in minute ventilation or its components, arterial oxygen saturation, heart rate, and oxygen consumption. End tidal carbon dioxide was lower than 40 mmHg before the addition of external breathing resistance, indicating that subjects generally hyperventilated when presented with the mouthpiece. However, end tidal carbon dioxide did not change with increasing resistance, suggesting that the addition of up to 20 cmH_2_O/L/sec external resistance did not limit the ability to maintain ventilation.

**Table 2:** Effect of external breathing resistance on cardiorespiratory variables

	Baseline	2 cmH_2_O/L/sec	4 cmH_2_O/L/sec	8 cmH_2_O/L/sec	12 cmH_2_O/L/sec	16 cmH_2_O/L/sec	20 cmH_2_O/L/sec
External inspiratory resistance							
Minute ventilation (L/min)	11.0 ± 3.0	9.8 ± 3.1	10.9 ± 2.7	15.8 ± 17.1	11.4 ± 3.5	10.3 ± 3.2	10.4 ± 3.0
Frequency (breaths/min)	14 ± 4	13 ± 3	13 ± 3	12 ± 3	13 ± 4	12 ± 4	13 ± 4
Expiratory time (%)	54 ± 6	48 ± 5	45 ± 7	45 ± 6	47 ± 6	43 ± 8	46 ± 8
SpO_2_ (%)	97 ± 2	97 ± 1	97 ± 2	97 ± 2	98 ± 1	98 ± 1	97 ± 1
Heart rate (beats/min)	63 ± 10	62 ± 11	62 ± 8	61 ± 8	61 ± 9	62 ± 11	62 ± 9
ETCO_2_ (mmHg)	35 ± 6	35 ± 6	35 ± 6	35 ± 6	35 ± 6	36 ± 5	36 ± 5
VO_2_ (L/min)	0.52 ± 0.3	0.54 ± 0.3	0.54 ± 0.3	0.61 ± 0.3	0.54 ± 0.3	0.56 ± 0.3	0.52 ± 0.3
External expiratory resistance							
Minute ventilation (L/min)	11.0 ± 3.3	9.0 ± 4.3	9.5 ± 3.4	9.1 ± 3.6	9.5 ± 5.2	9.1 ± 4.2	9.2 ± 4.67
Frequency (breaths/min)	15 ± 3	12 ± 3	13 ± 3	13 ± 3	12 ± 3	11 ± 3	11 ± 3
Expiratory time (%)	50 ± 7	51 ± 5	56 ± 5	52 ± 5	51 ± 7	55 ± 5	55 ± 4
SpO_2_ (%)	97 ± 1	98 ± 2	97 ± 1	97 ± 2	97 ± 1	98 ± 1	98 ± 1
Heart rate (beats/min)	64 ± 17	61 ± 10	59 ± 9	61 ± 10	61 ± 10	61 ± 10	62 ± 11
ETCO_2_ (mmHg)	37 ± 3	30 ± 4	33 ± 4	33 ± 4	33 ± 4	33 ± 4	34 ± 4
VO_2_ (L/min)	0.52 ± 0.3	0.51 ± 0.3	0.48 ± 0.3	0.51 ± 0.3	0.49 ± 0.3	0.49 ± 0.3	0.47 ± 0.3
Combined external resistance							
Minute ventilation (L/min)	9.7 ± 2.1	10.8 ± 3.7	9.2 ± 3.3	9.8 ± 2.4	9.6 ± 2.8	10.0 ± 2.1	9.4 ± 2.9
Frequency (breaths/min)	13 ± 2	13 ± 3	13 ± 3	11 ± 3	11 ± 3	11 ± 3	10 ± 4
Expiratory time (%)	50 ± 9	46 ± 5	51 ± 6	51 ± 6	51 ± 6	48 ± 7	49 ± 8
SpO_2_ (%)	97 ± 2	97 ± 2	97 ± 1	97 ± 1	97 ± 1	97 ± 1	97 ± 1
Heart rate (beats/min)	63 ± 12	63 ± 10	61 ± 10	65 ± 1	60 ± 8	63 ± 9	60 ± 17
ETCO_2_ (mmHg)	33 ± 13	31 ± 13	31 ± 12	32 ± 13	32 ± 13	35 ± 13	34 ± 13
VO_2_ (L/min)	0.52 ± 0.3	0.47 ± 0.6	0.44 ± 0.9	0.44 ± 0.6	0.44 ± 0.6	0.43 ± 0.6	0.40 ± 0.6

Values are mean ± SD. SpO_2_, arterial oxygen saturation; ETCO_2_, end tidal CO_2_; VO_2_, volume of oxygen uptake.

## Relationship between esophageal pressure and PFO patency

**Figure 2:**
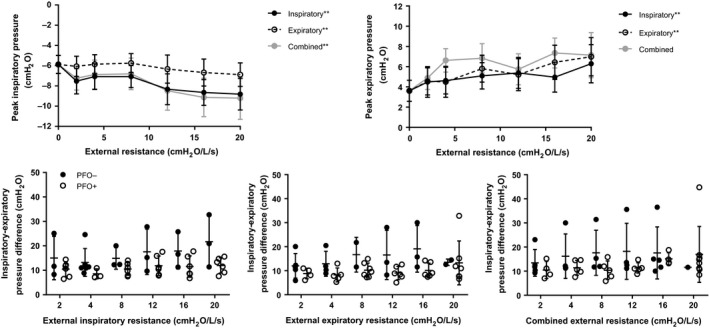
Top: Relationship between peak inspiratory and expiratory esophageal pressure and external resistance. Peak inspiratory pressure generally fell and expiratory pressure increased as a function of Increasing external resistance (**P < 0.05). Bottom: The peak inspiratory‐expiratory pressure difference was not difference between individuals with a recruited PFO (PFO ^+^) and a closed PFO (PFO ^−^). There were also no differences in peak inspiratory and expiratory pressures between PFO ^+^ and PFO ^−^ individuals (not shown).

For the inspiratory, expiratory and combined external resistance trials, individual variability was the most important predictor of the maximal and minimal esophageal pressure, and the maximal‐minimal pressure difference (*P* < 0.001 for all trials). Both maximal and minimal esophageal pressures were related to increasing external resistance for all three trials, with the exception of maximal pressure during the combined external resistance trial (Fig. 2 and summarized in Table 3). Participants did not alter tidal volume in response to resistance (Table 2). Therefore, consistent with the Ohmic relationships between pressure, flow and resistance, participants generally responded to increased resistance by generating more extreme pressures. For both external inspiratory, expiratory and combined resistance trials, there was no relationship between esophageal pressure and whether the PFO was recruited (Fig. 2).

**Table 3:** Significance of relationship (*P*‐value) between the changes in esophageal pressure and external breathing resistance

	Peak expiratory pressure	Peak inspiratory pressure	Expiratory‐inspiratory pressure difference
External inspiratory resistance	0.013	0.001	<0.001
External expiratory resistance	0.004	0.031	<0.001
Combined external resistance	0.068	<0.001	<0.001

Values are expressed as means ± the standard deviation. Inspiratory PES, peak inspiratory esophageal pressure; expiratory PES, peak expiratory esophageal pressure; HR, heart rate; SpO2, peripheral oxygen saturation;$\dot{V}$O_2_, oxygen consumption; $\dot{V}$E, minute ventilation; f, breathing frequency.

**Figure 3:**
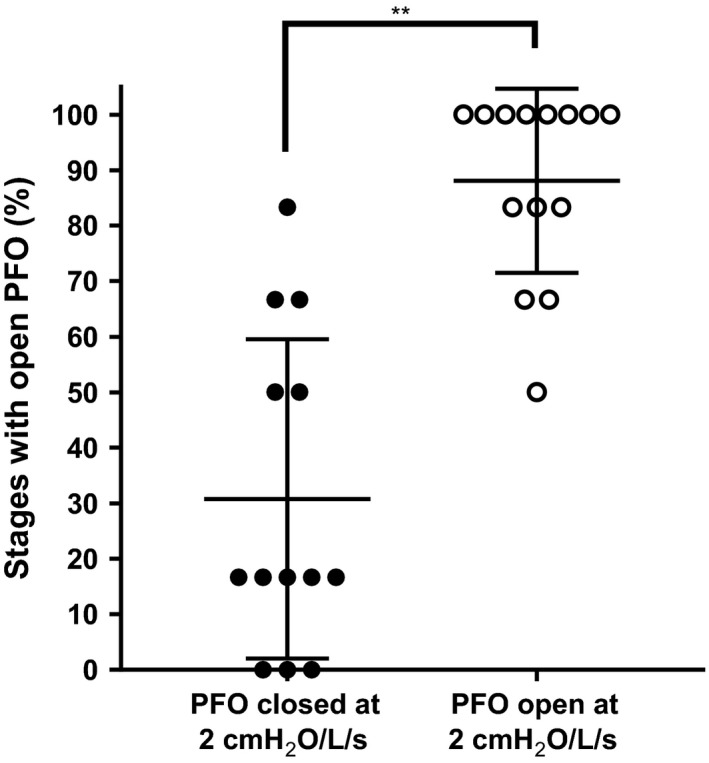
Comparison of the percent of stages with a recruited patent foramen ovale (PFO) between trials where individuals had their PFO closed at 2 cmH_2_O (closed circles) or open (open circles). Trials where the PFO was open at 2 cmH_2_O had a greater number of stages with a recruited PFO compared to trials where the PFO was closed (**P < 0.05 from repeated measures ANOVA).

We noted a tendency for particular individuals to rarely (11–44% of stages) or frequently (61–94% of stages) recruit their PFO in response to external resistance (Table 4). We asked whether recruitment of the PFO at 2 cmH2O/L/sec predicted whether individuals would rarely or frequently recruit. After considering the individual and external resistance type (inspiratory, external, and combined) as random, repeated factors, whether the PFO was observed at 2 cmH_2_O/L/sec predicted the number of stages with a recruited PFO (*P* = 0.004) (Fig. 3).

**Table 4:** Patent foramen ovale recruitment by stage for inspiratory, expiratory, and combined external resistance trials

Subject	External inspiratory resistance (cmH_2_O/L/min)								External expiratory resistance (cmH_2_O/L/min)									Combined external resistance (cmH_2_O/L/min)							Overall PFO+ Stages (%)
	0	2	4	8	12	16	20	PFO+ Stages (%)	0	2	4	8	12	16	20	PFO+ Stages (%)	0	2	4	8	12	16	20	PFO+ Stages (%)	
1								0							√	16							√	16	11
2						√		16					√			16							√	16	17
3		√		√	√	√	√	83			√	√		√		50								0	44
																									
4				√				16		√	√	√	√	√	√	100		√	√	√	√	√	√	100	61
5		√			√	√	√	66				√		√	√	50			√	√	√		√	66	61
6		√	√	√	√	√	√	100		√	√	√	√		√	83			√	√	√	√	√	83	89
7		√	√	√	√	√	√	100		√	√	√	√	√	√	100		√				√	√	50	83
8		√		√	√		√	66				√	√	√	√	66		√	√	√	√	√	√	100	78
9		√	√	√	√	√	√	100		√	√	√	√	√	√	100		√	√	√	√		√	83	94

√ indicates that a patent foramen ovale was visualized during that stage.

## Discussion

The primary aim of this study was to determine whether external breathing resistance enhances PFO recruitment. Consistent with our previous study investigating the impact of inspired oxygen concentration and position on PFO recruitment (Moses et al. 2015), we observed a high frequency of PFO recruitment at all levels of inspiratory, expiratory, and combined external resistance. We hypothesized that the more extreme positive and negative intrathoracic pressures necessary to maintain ventilation with increasing external resistance would relate to PFO recruitment. More extreme intrathoraic pressure could influence the pulsatile nature of venous return, and thereby right atrial pressure, via (1) an enhanced respiratory pump mechanism upon the initiation of inspiration or (2) collapse of the inferior vena cava and thoracic veins in response to markedly negative (i.e., the Starling resistor model) or positive intrathoracic pressure, with an transient increase in venous return during transition to the next phase of breathing (Olgiati et al. 1986; Robotham and Takata 1995; Harms et al. 1998; Kitano et al. 1999; Miller et al. 2005). Olgiati et al. (1986) found that the addition of inspiratory and expiratory resistance during resting breathing and exercise increased heart rate, and inspiratory resistance tended to increase stroke volume, albeit nonsignificantly. Contrary to our hypothesis, we did not find a relationship between recruitment of the PFO and intrathoracic pressure, estimated from measures of esophageal pressure. Although participants generally generated more extreme esophageal pressures with increasing external resistance in order to maintain ventilation, esophageal pressure was similar in individuals with and without a recruited PFO.

Patent foramen ovale is a common finding in adults (Fisher et al. 1995; Lovering et al. 2016). In human autopsy studies, the incidence of PFO decreases as a function of age (Hagen et al. 1984). Although there is a link between having a PFO and an increased risk of decompression illness, it is recommended that divers should only be screened for PFO after an episode of decompression illness (Sykes and Clark 2013). This is largely because PFO closure carries risk, not all divers with a PFO develop decompression illness, and the mechanisms underlying PFO recruitment are not well understood. One theory is that the development of gas emboli at the end of a dive occurs during a time when people might be performing Valsalva‐like maneuvers to clear their ears, move equipment, or strain to re‐enter a boat (Nishi 2003). In this study and our previous work, we found that the addition of only a minor amount of external resistance caused at least half of our participants to recruit their PFO, challenging the idea that a Valsalva is the impetus to open the PFO. Indeed, our data suggest that it is likely that the PFO is recruited throughout the dive in some individuals. Recruiting the PFO throughout the dive would allow a continuous bubble load to travel from the right to left atrium, compared to right‐to‐left passage during sporadic Valsalva and straining maneuvers.

Our data also suggest that there is substantial intrasubject variability in predicting who will open their PFO and this variability may depend on the individual's anatomy. Although our participants had similar grade PFOs by Valsalva screen, their recruitment was heterogeneous during the resistance challenge. Cartoni et al. (2004) characterized the PFOs of individuals with a previous episode of decompression sickness and found that they tended to have larger PFOs, but also tend to have more mobile flaps. Relating anatomic features of the PFO to risk require a transesophageal echocardiogram, which itself is an invasive procedure requiring sedation. Furthermore, while anatomic information is important, it does not provide information about the behavior of the PFO during physiologically important conditions. We propose that future studies should relate whether a diver recruits their PFO with 2 cm H_2_O external breathing resistance with incidence of decompression sickness. Our study requires only a transthoracic echocardiogram with intravenous agitated saline, provides physiological information about PFO recruitment, and may be a valuable screening tool in the future.

It is frequently recommended that in closed or semiclosed breathing apparatuses, where there is some latitude as to where resistance is applied, that the resistance be placed on the expiratory component of the apparatus (Warkander et al. 2001). Divers are better able to tolerate expiratory resistance during a dive. Adding inspiratory resistance of the same magnitude while submerged corresponds to greater dyspnea scores, greater end tidal CO_2_ levels, and lower maximum voluntary ventilation (Warkander et al. 2001). We found no difference between the effects of strictly inspiratory versus strictly expiratory resistance on the PFO. Therefore, although expiratory resistance may be better tolerated, there is an equal probability of PFO recruitment occurring regardless of where resistance is applied. Independent of decompression illness, recruitment of the PFO may have other important consequences for divers. Davis et al. (2015) found that individuals with a PFO have higher esophageal temperatures at rest and during exercise, possibly because the portion of blood that passes through the PFO is not cooled by the lungs. When immersed in 20°C, individuals with a PFO also maintain a higher body temperature (Davis et al. 2017), suggesting that blood flow through the PFO may be protective during a cold challenge. While we have focused on the passage of bubbles through the PFO, there may be other relevant physiological consequences of maintaining a patent foramen ovale during a dive that warrant exploration.

### Limitations and alternate interpretations

It is important to note that our study strictly examined the effects of external breathing resistance on PFO recruitment, in normobaric subjects in a dry environment. In the underwater environment, there are a multitude of additional variables to consider. In hyperbaria and cold‐water temperatures, the density of the gases that divers breathe increases (Marinovic et al. 2010). As a result, airway resistance is increased, further augmenting the work of breathing in divers who are already breathing against external resistance. Additionally, hyperbaric conditions, cold‐water temperatures, and immersion, all promote translocation of blood to the thorax, increasing central venous pressure (Boussuges et al. 2006; Marinovic et al. 2010). Indeed, the effects of increased central venous pressure may augment PFO recruitment created by breathing against resistance. While the aforementioned variables support that the underwater environment may further facilitate PFO recruitment in the face of external breathing resistance, there are also factors that may decrease PFO recruitment while diving. Translocation of blood to thorax and peripheral vasoconstriction both increase the left ventricular afterload (Marabotti et al. 2009; Marinovic et al. 2010). Increases in left ventricular and atrial pressures may create a left‐to‐right atrial pressure gradient that would oppose right‐to‐left flow through the PFO. Furthermore, the hydrostatic pressure imposed upon a diver at great depths may reduce the volume of the thoracic cavity (Marabotti et al. 2008). The reduction in thoracic volume combined with the translocation of blood to the thoracic cavity may hinder diastolic filling, and thus ventricular preload. Reductions in preload may inhibit the effects breathing resistance has on PFO recruitment. Future studies will be required to individually examine the effect of hyperbaria, temperature, and immersion on the PFO. However, our results strongly suggest that even a minimal amount of breathing resistance is sufficient to cause right‐to‐left bubble passage via the PFO.

In this study, we narrowly defined our subject population during screening to include only individuals with a Valsalva‐recruitable PFO in order to minimize confounding by other relevant cardiopulmonary structures. No effort was made to time the injection of contrast with the respiratory duty cycle, but participants were coached to relax and breathe normally during the non‐Valsalva portion of the screening. We excluded subjects with late appearing contrast without Valsalva because the transit route of the contrast may be complicated. Elliott et al. (2013) demonstrated that 28% of subjects demonstrate delayed contrast passage, which may be explained by transpulmonary passage. Because it would be difficult to separate PFO passage from transpulmonary passage, we excluded these participants. We also excluded participants that had passage in <3 cardiac cycles without Valsalva because it could be difficult to differentiate a large PFO from an atrial septal defect.

We did attempt to relate the magnitude of external resistance with semiquantitative measures of right‐to‐left bubble passage using a previously described bubble score (Moses et al. 2015), but did not find a relationship. We speculate that this is because our population tended to have only moderate baseline bubble scores (2–4, with only one subject scoring 4) and that we may not have enough variability in the baseline PFO size and large enough sample size to make these comparisons. Future studies that seek to address this issue should consider a larger number of subjects with greater variability in baseline PFO score. Additionally, we should note that we evaluated bubble passage across the PFO and not necessarily blood flow. There may be blood flow across the PFO even when there is not bubble passage. Still, while we believe it to be most relevant to divers to determine whether bubbles traverse the atrial septum, future studies relate bubble passage with whether there is blood flow crossing the PFO (Hlastala 1984; Mager et al. 2002).

By design, we chose not to standardize the inspiratory/expiratory duty cycle in order to emulate how divers realistically breathe in response to the resistance provided by SCUBA regulators, although participants in our study did not alter tidal volume or frequency in response to external breathing resistance. Therefore, they effectively maintained the respiratory duty cycle. Divers typically respond to external breathing resistance by lengthening the phase of respiration with the added resistance. Prolonging the expiratory portion of the duty cycle results in decreased mean airflow over time, and thus minimizes the work of breathing in the face of external resistance (Olgiati et al. 1986). In order to maintain minute ventilation, the diver would need to further shorten the inspiratory phase, which could collapse the thoracic veins and cause more pulsatile venous return (Olgiati et al. 1986). It is therefore possible that we might observe a different pattern of PFO recruitment if we had studied trained divers. Because of the design of our resistors, we placed the pneumotach on the expiratory side of the two‐way nonrebreather valve. Therefore, we do not know how ventilation changed with respect to lung volume during the different trials.

## Conclusions

In summary, our findings show that breathing resistance, similar to the levels contained in SCUBA regulators, causes right‐to‐left flow via the patent foramen ovale. In particular, the recruitment of the PFO with a small amount of external breathing resistance predicts the likelihood of patency at high breathing loads. Whether this is a valuable screening tool to predict decompression illness risk remains an important question for future study.
